# Active tactile sensibility of three-unit implant-supported FPDs versus natural dentition

**DOI:** 10.4317/jced.55748

**Published:** 2019-07-01

**Authors:** Ramin Negahdari, MohamadAli Ghavimi, Milad Ghanizadeh, Sepideh Bohlouli

**Affiliations:** 1 Assistant Professor, Department of Prosthodontics, Faculty of Dentistry, Tabriz University of Medical Sciences, Tabriz, Iran; 2 Assistant Professor, Department of Oral and Maxillofacial Surgery, Faculty of Dentistry, Tabriz University of Medical Sciences, Tabriz, Iran; 3 Postgraduate Student of Oral and Maxillofacial Surgery, Department of Oral and Maxillofacial Surgery, Faculty of Dentistry, Tabriz University of Medical Sciences, Tabriz, Iran; 4 Assistant Professor, Department of Oral Medicine, Faculty of Dentistry, Tabriz University of Medical Sciences, Tabriz, Iran

## Abstract

**Background:**

Splinting of the implants might improve the active tactile sensibility (ATS) of the pontic area due to cumulative effect of Osseo perception of two retainers; on the other hand, due to the lack of any supporting implant in the axis of occlusal force for the pontic area, ATS might be lower for this portion of FPDs. we evaluated the active tactile sensibility of natural teeth and three-unit implant-supported FPDs.

**Material and Methods:**

The ATS of posterior 3-unit implant-supported FPD and contralateral teeth was measured in 50 patients, in a random order blinded to patients and assessor, carried out at two sessions. Based on the experimental range of 0 to 70 um, the sigmoid shape of psychometric curve was estimated to locate the 50% values as the ATS thresholds for each tooth or implant. Data were analyzed using unpaired t-tests.

**Results:**

The ATS of the teeth and implants differed significantly and compared to teeth, implants exhibited significantly higher ATS thresholds in all the groups. The results of independent t-test showed the highest difference in the means of ATS between the pontic and the first molar tooth. Based on the equivalence testing approach, the 95% CIs indicated that the differences were clinically significant only in the Pontic/First Molar group.

**Conclusions:**

In multi-unit implant-supported prostheses the tactile perception of the prosthesis that are placed on fixtures is similar to the natural teeth. In pontic areas there are significant statistical and clinical differences, with much lower tactile sensibility in pontics compared to the natural teeth.

** Key words:**Active tactile sensibility, dental implants, interdental perception, osseoperception.

## Introduction

The periodontal ligament sends tactile stimuli to the central nervous system (CNS) via its mechanoreceptors. These receptors exhibit sensitivity to direction and magnitude of loads applied and mediate the adaptation of centrally generated biting and chewing patterns by transmission of afferent information on the magnitude and direction of occlusal load (1). When an implant replaces a natural tooth, the periodontal ligament disappears; therefore, the periodontal ligament proprioceptive function cannot mediate sensorimotor regulation of masticatory functions (2).

However, a phenomenon referred to as Osseo perception is an important step toward functional and physiologic integration of implants into the masticatory system (3,4). It has been proposed that Osseo perception might be induced by mechanoreceptors in the remote nerve endings, periradicular tissues of the antagonist teeth, cortical synaptic remodeling in the brain, or possibly from the innervation of peri-implant tissues (3-5). This reconstruction of the sensory-motor function which is mediated by the implant might help achieve a more natural oral function, yielding important clinical outcomes (6). A highly sensible implant night result in the recovery of proper sensory-motor control mechanisms, improving masticatory efficacy, inhibitory reflex responses in masticatory muscles (preventing traumatic occlusion) and sensory discriminative potentials, thus decreasing the risk of overloading the implants and remaining natural teeth (3,7,8).

Several studies have evaluated the tactile sensibility of dental implants and most of them have used passive assessment of tactile sensibility which applies certain loads on implants (5,9-13). Active tactile sensibility evaluation, however, results in better simulation which actually happens in clinical conditions (3). Passive tactile perception is carried out by the application of certain loads on tooth/implant and records minimum detectable force by patient. Active tactile sensibility is measured by placing foreign bodies between tooth/implant and recording the minimum detectable thickness (3,5).

A meta-analysis by Higaki *et al.* showed that the tactile sensibility of an implant was significantly lower than that of a natural tooth (higher foil-thickness discrimination) (14). In all the studies on this issue, tactile sensibility was compared between teeth/implant-supported single-unit or implant/tissue-supported removable prosthesis (4,5,9-13,15,16). In a previous study, we explored the difference between the ATS of a natural tooth and single-unit implant-supported crowns in a split-mouth pattern. The results indicated that the tactile sensibility of natural teeth was higher than the implants (4).

In the implant-supported FPDs, implants are splinted together with the pontic area between them; the ATS of the pontic area might be higher or lower than that of the retainers. Splinting of the implants might improve the ATS of the pontic area due to cumulative effect of Osseo perception of two retainers; on the other hand, due to the lack of any supporting implant in the axis of occlusal force for the pontic area, ATS might be lower for this portion of FPDs. To clarify this, as continuation of the previous study (4) and regarding the fact that tactile sensation of multi-unit implant-supported FPDs has not been studied yet, in this study we evaluated the active tactile sensibility of natural teeth and three-unit implant-supported FPDs.

## Material and Methods

-Study design and patients

This split-mouth, double-blind, randomized clinical trial was performed on 50 patients with 50 posterior 3-unit implant-supported FPD with second premolar and second molar implant retainers (2013‒2015). The inclusion criteria consisted of patients’ willingness to participate, a proper occlusion of a 3-unit implant-supported FDP with a porcelain fused-to-metal crown and its antagonist teeth on one side and occlusion of the corresponding pair of antagonist natural teeth (second premolar, first and second molar) on the contralateral side, a minimum of 6 months of successful implant function in a competent occlusion based on clinical and radiographic examinations, and absence of any root canal treatments, coronal restorations, or any pathologic mobility of the natural teeth, any bone loss around the teeth, and any evidence of malocclusion, any premature or open interocclusal contacts, as well as any signs/symptoms of TMJ disorders. Five subjects were excluded from the study due to insufficient compliance or lack of stable occlusal contacts. Ninety implants used in this trial were of ITI Bone fit implants (Strauman GmbH, Freiburg, Germany).

-Ethical Approvals

The protocol of the study was approved by the internal review board of the university in accordance with the Declaration of Helsinki, and signed written consents were taken from the patients prior to the study.

-Data collection

Despite the fact that the protocol of the study was explained to the patients in detail, the aim of the study was not described in order to prevent a bias in their responses. Both the patient and operator were unaware of the aims of the study to avoid the reporting bias (double-blind). The subjects were instructed to avoid eating or chewing 1 hour prior to the study (17). The patients were seated in a semi-supine position on a dental unit in a quiet room, wearing eye-pads (6). They were asked to close their eyes during the experiments. Proper and stable occlusal contacts on the involved teeth and prostheses were confirmed by examining with a 15-um thin articulation band (Arti-Foil, Bausch KG, Cologne, Germany) in maximum intercuspation. The occlusal contacts were initially marked with the articulating paper (Arti-Foil, Bausch GmbH, Germany). Industrially manufactured 24-karat gold foils were used (Mitotoyo, Japan), which measured 20 to 70 um in thickness, 3 mm in width and 3 mm in length. They were held by a needle holder. Before asking each patient to bite on the foil, the foil was molded on the marked occlusal surface of the mandibular tooth/prosthesis. Therefore, after each experiment, it was distorted and disposed of. The thickness of each foil was tested 5 times for two retainers and pontic area separately, based on a computer-generated random order unknown to the observer and the patient. The subjects were instructed to report the presence or absence of the foil after occluding. Both the implant and control sides were examined.

In order to include the 0-um foil thickness in the model, and exclude response bias/patient guessing, there was a mock trial in each row during the examination of each side (five trials per each tooth at each session). The patients had been informed of this before the study procedures began.

Subjects claiming to sense a placebo (null) foil on both sides were to be excluded and no subject met this exclusion criterion. Patient response to the placebo trials (0-um thickness) were also used in estimating the psychometric curve. The tests were repeated for each patient after at least 1 week.

Estimation of ATS by drawing the psychometric curve

Based on the responses provided by each patient on each side, to the range of 0‒70-um thicknesses, a sigmoid psychometric curve (fitted on the cumulative Weibull distribution) was computed for each tooth side in all the patients. Based on the estimated function of the psychometric curve, the foil thickness at which the 50% value stood was located as the ATS threshold. The 50% values were recorded for each tooth and implant in each subject at each interval.

-Statistical analysis

Based on a pilot study on 10 subjects, the sample size was calculated at 50 implants and 50 teeth to obtain a test power of >0.90 (d = 8 um, SD = 8 um) at a 0.05 level of significance. The results of the pilot study were included in this research. Descriptive statistics were reported for the ATS thresholds (the 50% values) in all the sample at each session. There was a high rate of intra-observer agreement between the values obtained at the two intervals (Cohen’s Kappa >0.92, *P* = 0.0001). Tactile perceptions (the 50% values) of the teeth were compared with the ATS values of the implants using unpaired samples t-test. Statistical significance was set at 0.05.

-Equivalence testing 

In order to investigate the clinical significance semi-objectively, 95% confidence intervals (CIs) were calculated for ATS differences between the teeth and implants. The CIs were compared with an 8-um thickness as the margin of clinical equivalence, similar to studies by Enkling *et al.* (5,15). Only when both CI bounds were simultaneously below and beyond the range of -8 to 8 um, it could be certainly inferred (at 95% CI) that the difference between the tooth and implant varied from this margin and therefore might be clinically significant. Otherwise, the result would be inconclusive in terms of practical significance.

## Results

Fifty patients with 50 posterior 3-unit implant-supported FPD with second premolar and second molar implant retainers were evaluated in this study and none was excluded due to falsely reporting perception of mock trials on both sides. Five subjects were excluded from the study due to insufficient compliance or lack of stable occlusal contacts. The results indicated that the ATS of the teeth and implants differed significantly and compared to teeth, implants exhibited significantly higher ATS thresholds in all the groups. In addition, the results of independent t-test showed the highest difference in the means of ATS between the pontic and the first molar tooth and the minimum difference between the anterior retainer of the implant and the second premolar tooth ( Table 1).

**Table 1:**
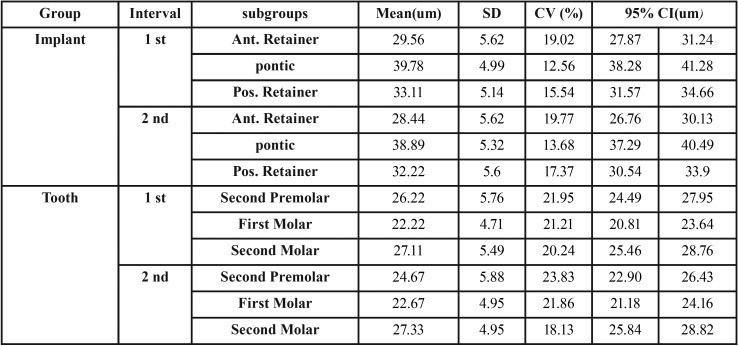
Active Tactile Sensibility Thresholds for the Implants and Teeth.

Based on the equivalence testing approach, the 95% CIs indicated that the differences were not clinically significant in any of the groups except for the Pontic/First Molar group ( Table 2).

**Table 2:**
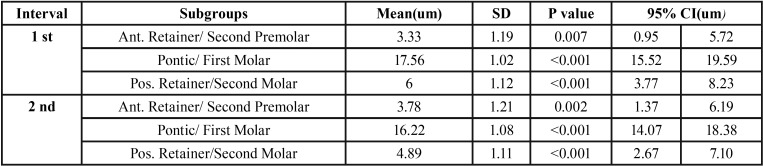
Active Tactile Sensibility Differences between the Implants and Teeth.

## Discussion

Elimination of PDL proprioceptive fibers during tooth extraction(6,18) compromises sensory-motor control mechanisms. Rehabilitation of the sensory-motor interaction with the use of implants might help achieve a more natural oral function, with important clinical outcomes (6). A properly tactile-sensible implant is conducive to appropriate sensory-motor control, improving masticatory efficacy, inhibitory reflex responses in masticatory muscles to prevent traumatic occlusion, and sensory discriminative potentials; therefore, it can decrease the risk of overloading the remaining teeth and implants (3,7,8). Therefore, it is very important to assess the ability of implants to discern fine tactile stimuli and since the tactile sensation of multi-unit implant-supported FPDs has not been evaluated yet, this study was undertaken to evaluate the active tactile sensibility of natural teeth and three-unit implant-supported FPDs. Of the three studies that have compared implant and tooth ATS, one reported statistical significance only (8), while two used only the clinical significance with the equivalence testing approach, not the hypothesis testing (5,15). In this study both methods were used to improve the capacity to compare the results.

The results of this study showed that compared to natural teeth, implants exhibited significantly higher ATS thresholds in all the groups.

Such difference in the mean ATS of the pontic and the first molar tooth was statistically and clinically significant. However, despite the statistically significant difference between the mean ATS of the anterior and posterior implant abutment teeth and the second premolar and second molar, the difference was not clinically significant. Different studies have reported an ATS threshold 3‒6 times that of natural teeth for implants (3,6,7,19). On the other hand, some researchers have reported ATS values of 50‒100 µm for implants (19,20). However, although the sensibility of implants is much lower than that of natural teeth, dental implants have succeeded in satisfying patients in this respect (11,19).

Similar to the results of present study, Batista *et al.* have reported a significant but minor difference in the discriminative ability between the teeth and implant(21). Enkling *et al.*, too, reported an approximate difference of 2-3 µm and concluded that the tactile sensibility of single-unit implants is comparable to that of natural teeth (3).

In a study by Kazemi *et al.*, too, despite a minor statistical difference, there was no clinical difference in ATS between the natural teeth and single-unit implants. In that study the ATS threshold in single-unit dental implants was higher than that in natural teeth (4). In an animal study, Ysander *et al.* showed that implant materials are surrounded by the nerve fibers remaining from the periodontal tissues of the extracted tooth at implant‒bone interface (22). However, since the density of these nerve fibers decrease around the dental implants with an increase in bone contact rate, it is expected that there will be a different tactile perception around the implants with a change in bone apposition rate in different areas of the jaw (22). This might explain why differences in tactile perception are reported in different areas of the jaws, with a significant difference in tactile perception at pontics, where there is no contact with bone.

In another study, Griezni *et al.* compared the passive tactile sensibility of dental implants and natural teeth and concluded that to achieve touch sensation in dental implants, it is necessary to apply a greater force (9). Such a difference between active and passive thresholds might be attributed to several different receptor groups that respond to active testing while the passive methods selectively stimulate periodontal ligament receptors (6).

Although similar to the present study, several studies have shown ATS values similar to natural teeth in implant-supported prostheses that are placed on fixtures and are directly related to the jaw bone, in the present study lower ATS values were reported in the pontic areas of implant-supported prostheses. Therefore, it is very important to adjust the occlusal relationships of implant-supported prostheses in the pontic areas because some occlusal interferences in the pontic areas of implant-supported prostheses that are at a low level relative to the ATS of the areas will not be perceived by the patients and relying on the patient’s tactile perception during occlusal adjustment will possibly result in occlusal overload and problems resulting from it. It appears during the process of occlusal adjustment of implant-supported prostheses it is more logical to evaluate objective evidence and rely on the clinical judgment of the dentist, rather than the patient’s tactile perception. On the other hand, based on the results of the present study, it is necessary to include a greater number of implants in the treatment plan and decrease the number of pontic in order to increase the patients’ tactile perceptions and their satisfaction in implant-supported prostheses.

-Limitations and strengths 

This study was limited by some factors. One limitation was the difficulty of standardization of the intensity of static occlusal contact in the oral cavity, which proved impossible. Nevertheless, the intra-individual comparison in this study decreased the impact of this factor. Some factors increased the reliability of the findings. Unlike previous studies, low-hardness gold foils were used in this study, which were easily adjusted. Therefore, they could be burnished and might been less likely to click in ears through bones. Enkling *et al.* (3,5,19) masked this click sound by transmitting high-pitched noises into the patients’ ears. Although this method succeeded in eliminating the sound of biting on the foils, it might have in turn served as a strong distractor and confounder (6), making the patients lethargic or decreasing their focus on tactile perception and the commands.

This study was the first study to adjust the foils on the occlusal surface before biting. Use of unadjustable foils does not guarantee a cusp-to-fossa contact; therefore, it cannot represent mouth closure in maximum intercuspation. In addition, proper occlusal contacts were assessed on both sides in this study as an inclusion criterion, which was used in only one previous study on teeth but not on implants (19). Furthermore, foil temperature was controlled at 25°C in this study because it might affect the tactile sensitivity via influencing pulp receptors (20). Furthermore, burnishing the foil on the tooth increased its temperature to a level higher than the oral cavity temperature (3,5,15,19). Enkling *et al.* instructed patients to operate the right and left mouse keys to signal the response, which required some manual skills and intelligence (5,15,19). However, in the present study, the patients were asked to report the sense of contact by raising a hand, which proved more convenient and less confusing. Based on a previous study, the 50% value was found in this research for each tooth or implant using the psychometric function based on patient answers, as the most reproducible method (3), instead of finding the thinnest detectable foil or the 80% value. Another advantage was the split-mouth design of this study because it was possible to eliminate a high rate of inter-individual variations. An important consideration in clinical significance is the subjective nature of the subject. We tried to integrate it with the semi-objective method of equivalence testing by adopting an objective threshold from previous studies (5,15). In this context, Enkling *et al.* (3,5,15,19) considered a minimum thickness detectable, below which no subjects were able to feel the foils. However, this minimum limit employs the abilities of subjects with the best perception capacity, ignoring less precise sensitivities. Therefore, in future studies the mean thickness of the thinnest foils felt by different subjects should be used. Consistent with studies by Enkling *et al.* (3,5,15,19), and contrary to some other studies (21), we applied the foils in a random manner to prevent patients’ learning that might have led to false positive responses in detecting very thin foils. Both the patient and operator were unaware of the aims of the study to avoid the reporting bias. Moreover, contrary to previous studies, in this study, each patient was examined twice at two intervals to decrease the effect of psychophysical status on the responses provided and promote the generalizability of the results. Carrying out the study procedures in two sessions, and the smaller number of test foils at each trial made each session as brief as possible (20-30 minutes in this study versus about 2 hours in another study) (5). This helped maintain patient focus and concentration during the test. Another advantage of this design compared to other studies (3,5,15,19,21) was that the subjects had been asked to avoid chewing on gums or foods before examinations in order to eliminate the possibility of receptor numbness (17). A large sample size, various types/brands of implants with different surface types and testing on different sites/genders can help promote the generalizability of the findings. However, no implants with machined or very rough surfaces were included, limiting the generalizability to rough surfaces. Some investigators have used smaller (21) or larger sample sizes (3,5,15,19). Only two of these sample sizes were determined based on power calculations to obtain a power of ≥0.8) (5,15). Although our sample size was smaller (n = 50 teeth and 50 implants), considering the high power of the study (>0.95) and exclusion of inter-individual variations, its size seemed sufficient. In this context, very large sample sizes and very high powers might increase false positive responses and should be avoided as far as possible. It is advisable to compare pairs of occluding teeth with occluding antagonist implants, rather than implant-tooth pairs, to decrease the role of remaining PDL on the implant-tooth side, which is considered a confounding factor. However, it is very difficult to find such patients, and this has not been possible in any of the previous studies, either. Furthermore, the results of this study clearly showed that even despite the partial presence of PDL, significant decreases in the ATS were detected. The significant results, very low variations, and the high intra-observer agreement confirm the sufficient power and proper control over the confounders in the present study.

## Conclusions

In multi-unit implant-supported prostheses the tactile perception is almost similar to that of the natural teeth in areas of the prosthesis that are placed on fixtures, when there is a direct relationship with the jaw bone; however, in pontic areas there are significant statistical and clinical differences, with much lower tactile sensibility in pontics compared to the natural teeth.
